# The Network Structure of Symptoms of the Diagnostic and Statistical Manual of Mental Disorders

**DOI:** 10.1371/journal.pone.0137621

**Published:** 2015-09-14

**Authors:** Lynn Boschloo, Claudia D. van Borkulo, Mijke Rhemtulla, Katherine M. Keyes, Denny Borsboom, Robert A. Schoevers

**Affiliations:** 1 Department of Psychiatry, Interdisciplinary Center Psychopathology and Emotion Regulation (ICPE), University Medical Center Groningen, University of Groningen, Groningen, The Netherlands; 2 Department of Psychology, University of Amsterdam, Amsterdam, The Netherlands; 3 Department of Psychiatry / Department of Epidemiology, Columbia University, New York City, New York, United States of America; Maastricht University, NETHERLANDS

## Abstract

Although current classification systems have greatly contributed to the reliability of psychiatric diagnoses, they ignore the unique role of individual symptoms and, consequently, potentially important information is lost. The network approach, in contrast, assumes that psychopathology results from the causal interplay between psychiatric symptoms and focuses specifically on these symptoms and their complex associations. By using a sophisticated network analysis technique, this study constructed an empirically based network structure of 120 psychiatric symptoms of twelve major DSM-IV diagnoses using cross-sectional data of the National Epidemiologic Survey on Alcohol and Related Conditions (NESARC, second wave; N = 34,653). The resulting network demonstrated that symptoms within the same diagnosis showed differential associations and indicated that the strategy of summing symptoms, as in current classification systems, leads to loss of information. In addition, some symptoms showed strong connections with symptoms of other diagnoses, and these specific symptom pairs, which both concerned overlapping and non-overlapping symptoms, may help to explain the comorbidity across diagnoses. Taken together, our findings indicated that psychopathology is very complex and can be more adequately captured by sophisticated network models than current classification systems. The network approach is, therefore, promising in improving our understanding of psychopathology and moving our field forward.

## Introduction

Current classification systems, such as the *Diagnostic and Statistical Manual of Mental Disorders* (DSM) or *International Classification of Diseases* (ICD), have greatly contributed to the reliability of psychiatric diagnoses and are highly valuable in clinical practice. These classification systems are based on traditional models of medical diseases, which assume that symptoms are indicators of a common cause (i.e., the underlying disease). In such a latent variable model, an underlying attention-deficit/hyperactivity disorder may lead to attention problems as well as hyperactivity/impulsivity. Similarly, depressed mood and insomnia may be caused by an underlying major depressive episode. This model implies that symptoms are psychometrically interchangeable [1,2] and summing sets of symptoms in order to establish psychiatric diagnoses would, consequently, be an efficient way of reducing measurement error [3].

However, psychiatric diagnoses, even within the same category, are believed to be heterogeneous, as patients differ substantially in, for example, the naturalistic course of their illness, their underlying biology and their response to treatment. In order to explain this heterogeneity, classification systems offer the opportunity to distinguish subtypes within a particular diagnosis based on clusters of symptoms. For example, persons with an attention-deficit/hyperactivity disorder can be divided into those with a predominantly inattentive subtype, a predominantly hyperactive/impulsive subtype, and a combined subtype; especially this combined subtype has shown to have a poor prognosis [4]. Similarly, within a major depressive episode, an atypical subtype can be distinguished based on specific symptoms such as hypersomnia and an increase in weight/appetite; patients with such an atypical subtype have more inflammatory and metabolic dysregulation than other depressed patients [5]. To further explain the heterogeneity across psychiatric patients, classification systems also offer the opportunity to establish multiple diagnoses in the same patient (i.e., comorbidity). For example, depressive disorders are highly comorbid with anxiety disorders, and vice versa [6], and especially patients with such a comorbid condition have a poor prognosis [7].

Symptomatic subtypes of psychiatric diagnoses have helped to explain a part of the heterogeneity within psychiatric diagnoses and suggest that it might be valuable to focus on even smaller entities within diagnoses; that is, the individual symptoms. This approach is promising, as recent research, mainly on major depressive disorder, showed that individual symptoms differed in both their risk factors [8,9] and consequences [10]. Interestingly, symptoms of a major depressive disorder also differed in their comorbidity patterns with other diagnoses [11]; for example, psychomotor agitation/retardation, and not any other symptom, was associated with generalised anxiety disorder, whereas feelings of worthlessness/guilt was associated with both nicotine and alcohol dependence. This suggests that some, but not all, symptoms of a particular diagnosis, account for the comorbidity patterns between diagnoses.

These findings indicate that individual symptoms have a *unique* role and are *not* interchangeable. The strategy of summing symptoms in order to establish diagnoses would, consequently, result in loss of information. Research may, therefore, benefit from a conceptualisation that more adequately captures the complexity of psychopathology [12].

Recently, a new conceptualization has been proposed, which ignores the overarching diagnoses and specifically considers individual symptoms and their associations. The approach is based on the theory of complex networks [13,14] and revolves around the idea that psychopathology results from the causal interplay between symptoms [1,2,15,16]. In a major depressive episode, for example, feelings of worthlessness may cause a depressed mood while insomnia is likely to cause fatigue. Causal links may be weaker or even absent for other symptoms within the same diagnosis; for example, an increase in appetite is unlikely to directly cause suicidal ideation.

By using time-series analyses on data of multiple assessments with short time intervals, the specific causal associations between symptoms can be identified [17–19]. This information can, then, be visualized into a directed network, in which symptoms are represented as nodes and the causal links as arrows. It is, however, not feasible to assess large sets of symptoms multiple times a day. As a first step in identifying those symptom associations that are of interest for further examination, it might be valuable to determine an undirected network based on cross-sectional data. The complex associations between symptoms can also be visualized into a network structure, but the direction of associations is unknown and, therefore, associations are represented as edges.

The advantage of the network approach is that it naturally accommodates the unique role of each of the individual symptoms. If associations between symptoms within the same diagnosis would differ, this implies that these symptoms are not interchangeable; the strategy of summing symptoms in order to establish diagnoses would, therefore, lead to loss of information. In addition, it is important to note that the network approach allows specific symptoms of one diagnosis to be related to specific symptoms of another [2,15,16]. As some diagnoses are based on similar symptoms (e.g., criteria for major depressive episode, dysthymia, mania or hypomania, generalized anxiety disorder and post-traumatic stress disorder all include sleep disturbances), these overlapping symptoms are likely to show strong associations. In addition, specific non-overlapping symptoms of different diagnoses may also be related. If only some, and not all, symptoms of a particular diagnosis show connections with some, but not all, symptoms of another diagnosis, this implies that the specific symptom pairs connecting the two diagnoses can account for their comorbidity; focussing on diagnoses instead of symptoms would, again, lead to loss of information.

So far, only few studies have examined the network structure of psychiatric symptoms and empirical work has considered only two diagnoses and included 20 symptoms at most [2,16]. To improve our understanding of psychopathology, it is essential to consider symptoms across a wide spectrum of psychiatric diagnoses and, therefore, we used data from a large community sample (National Epidemiologic Survey on Alcohol and Related Conditions, NESARC; N = 34,653) [20] including 120 psychiatric symptoms of twelve major DSM-IV diagnoses. Our research aimed to determine the empirical network structure of these symptoms, and we explicitly focussed on the associations of symptoms within the same diagnosis and associations of symptoms between diagnoses.

## Materials and Methods

### Study sample

Data were derived from the second wave of the National Epidemiologic Survey on Alcohol and Related Conditions (NESARC; 2004–2005), which surveyed 34,653 adults (≥18 years) in the United States [20]. All potential participants were informed in writing about the nature of the survey, the statistical uses of the survey data, the voluntary aspect of their participation, and the federal laws that secure the confidentiality of identifiable survey information. Only participants who gave written informed consent were interviewed. A detailed description of the study design and sampling procedures can be found elsewhere [20,21]. The research protocol, including informed consent procedures, received full ethical review and approval from the US Census Bureau and the US Office of Management and Budget.

### Assessment of psychiatric symptoms

The structured NIAAA Alcohol Use Disorder and Associated Disabilities Interview Schedule–DSM-IV Version (AUDADIS-IV) [22] was used to establish the presence or absence of 120 symptoms of twelve psychiatric diagnoses. The considered psychiatric symptoms generally refer to the individual DSM-IV criteria. However, some criteria are based on several symptoms with differential meanings. As these symptoms may also differ in their associations with other symptoms in the network, the criteria were disaggregated. Criterion A of a (hypo)manic episode is, for example, disaggregated into an elevated mood and an irritable mood. Similarly, criterion A4 of a major depressive episode is disaggregated into insomnia and hypersomnia.

Symptoms of major depressive episode, dysthymia, mania or hypomania, generalised anxiety disorder, social phobia, specific phobia, panic disorder, agoraphobia, alcohol abuse or dependence, and nicotine dependence were assessed with a timeframe comprising the three years prior to the interview. In addition, symptoms of attention-deficit/hyperactivity disorder during childhood and lifetime symptoms of post-traumatic stress disorder were established. We used data from the second wave of NESARC, as (most) symptoms were assessed in a more specific timeframe (i.e., past three years) than at the first assessment (i.e., lifetime timeframe). The assessment of recent symptoms limits the risk of recall bias and is more appropriate for a network approach in which symptoms are assumed to be causally related. In addition, symptoms of attention-deficit hyperactivity disorder and post-traumatic stress disorder were only assessed at the second wave of NESARC.

The AUDADIS-IV includes screening questions for all psychiatric diagnoses, except for attention-deficit/hyperactivity disorder. Symptoms of a particular diagnosis were, thus, only assessed in participants who answered ‘yes’ to that screening question and not in those who answered ‘no’. As implied by the skip logic, the skip-related missing values on the subsequent, non-screening questions were imputed with zeros, indicating absence.

For five diagnoses, this imputation strategy follows logically from the structure of the DSM; that is, a negative response to the first, core symptom (i.e., the screening question) logically implies a negative response to (most) other symptoms. If participants, for example, did not experience any fear of a specific situation (screening question; criterion A of specific phobia), this (non-existing) fear cannot be excessive or unreasonable (criterion C). Similarly, participants who were not exposed to a traumatic event (screening question; criterion A1 of post-traumatic stress disorder), logically have no recollections (criterion B1) or distressing dreams (criterion B2) of that (non-existing) event. The same holds true for most symptoms of social phobia, panic disorder, and agoraphobia and we, therefore, believe that our imputation strategy is sensible for these five diagnoses.

For two other diagnoses, the imputation strategy also seems valid, as participants with a negative response to the screening question are highly unlikely to have experienced any of the symptoms. For example, participants who did not use any alcohol in the last three years (screening question for alcohol abuse or dependence) are highly unlikely to experience any problems due to their drinking. Similarly, participants who smoked no more than 100 cigarettes in the past three years (i.e., no more than 1 cigarette per 10 days; screening question for nicotine dependence) are highly unlikely to experience any of the symptoms of nicotine dependence.

Taken together, data on symptoms of social phobia, specific phobia, panic disorder, agoraphobia, post-traumatic stress disorder, attention-deficit/hyperactivity disorder, alcohol abuse or dependence, and nicotine dependence are probably valid. We are, therefore, confident that for these 75 symptoms the presented network reflects the “true network”. However, the imputation strategy is questionable for the 45 other symptoms in the network, belonging to major depressive episode, dysthymia, mania or hypomania, and generalised anxiety disorder. For example, participants who did not report low mood and loss of interest (screening questions; criteria A1 and A2 of major depressive episode, respectively) could have experienced other symptoms of a major depressive episode, such as insomnia (criterion A4) and concentration problems (criterion A8). Our imputation strategy may have affected the network estimation and we have, therefore, performed a set of sensitivity analyses (see Results section). A complete overview of the screening questions and other questions is presented in S1 Table.

### Statistical analyses

To estimate the network structure of 120 psychiatric symptoms, our novel network analysis technique eLasso (package ‘IsingFit’ in R) [23] was used. This technique is based on Ising models as used in physics and combines *l*1-reguralized logistic regression [24] with Bayesian neighborhood selection [25–27] to identify symptom-symptom associations that define connections in the network. Based on the estimated connection strengths between all psychiatric symptoms, a weighted, undirected graph was visualized using package ‘qgraph’ in R [28]. Symptoms were represented as nodes and associations between them as edges. Green edges represented positive associations and red edges represented negative associations, while the thickness of edges indicated the strength of associations. The layout of the graph was based on the Fruchterman-Reingold algorithm, which iteratively computes the optimal layout so that symptoms with stronger and/or more connections are placed closer to each other [29].

## Results

### Study sample

In our sample of 34,653 adults, 58.0% were female and the mean age was 49.1 (SD = 17.3) years. S1 Table shows the prevalence rates (N, %) of all individual psychiatric symptoms.

### The general network structure

**Fig 1:**
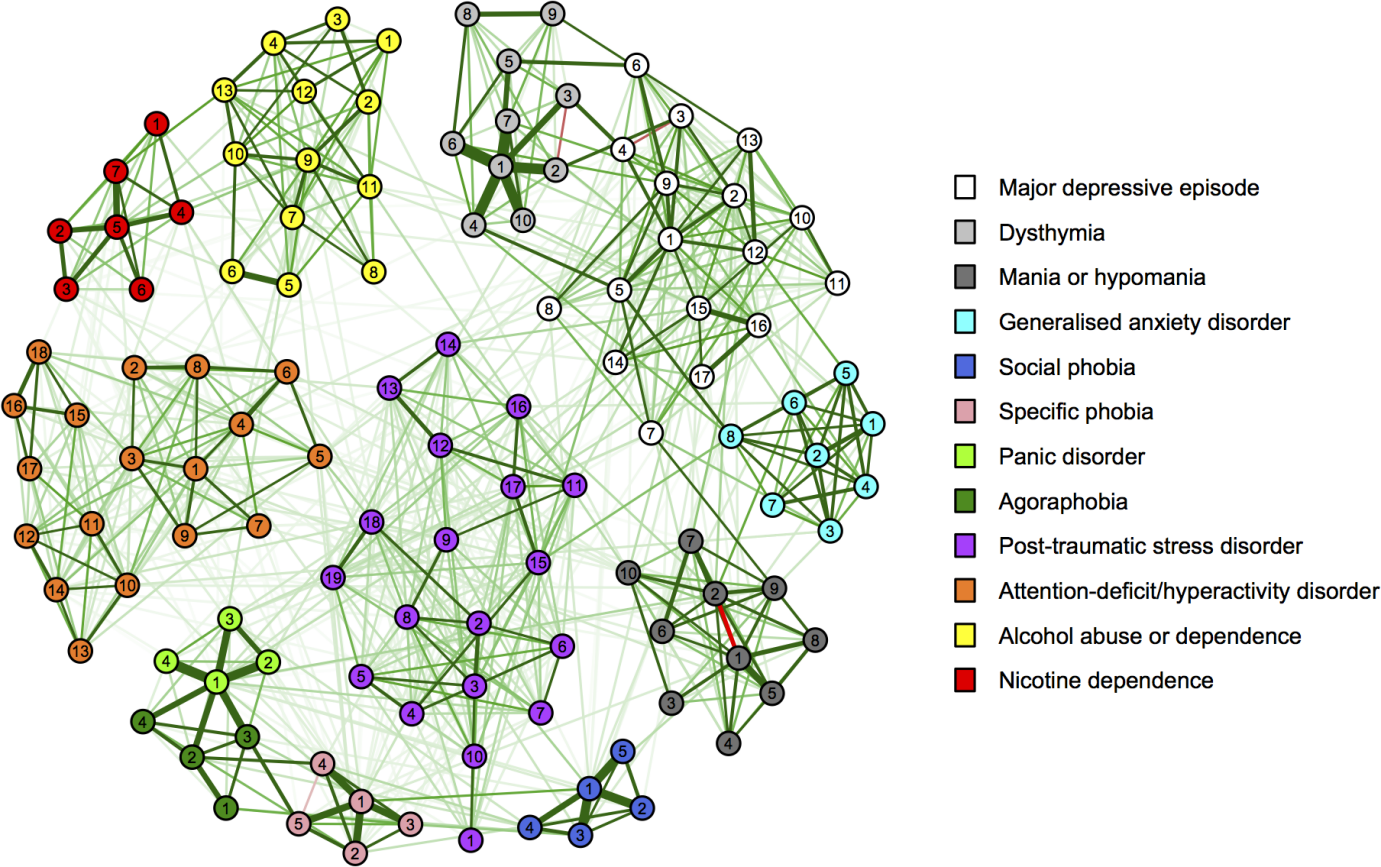
Empirical network of 120 psychiatric symptoms. Symptoms are represented as nodes and associations between them as edges. Node colours refer to the type of diagnosis and numbers refer to specific symptoms (see S1 Table). Green edges represent positive associations and red edges represent negative associations, while the thickness of edges represent the strength of associations.

The empirical network structure of the 120 psychiatric symptoms is presented in Fig 1, which is based on the estimated connection strengths between all 120 individual symptoms (see S2 Table). The network shows that all symptoms were connected, either directly or via other symptoms. It also had a high level of clustering (i.e., clustering coefficient = 0.45) and incorporated several clusters of highly connected symptoms, representing each of the separate psychiatric diagnoses. These clusters of symptoms showed strong interconnections and, consequently, path lengths from one symptom to another are short (i.e., few intermittent nodes; average shortest path length = 2.48). An overview of the connections of symptoms within and between diagnoses is presented in Table 1.

**Table 1:** Overview of the number of connections within and between diagnoses.

		MDE	Dys	Man	GAD	Soc	Spe	Pan	Ago	PTSD	ADHD	Alc	Nic
MDE	%	**68.4%**											
	N	**93/136**											
Dys	%	5.9%	**75.6%**										
	N	10/170	**34/45**										
Man	%	8.8%	0.0%	**88.9%**									
	N	15/170	0/100	**40/45**									
GAD	%	5.9%	2.5%	2.5%	**100%**								
	N	8/136	2/80	2/80	**28/28**								
Soc	%	8.2%	0.0%	6.0%	2.5%	**90%**							
	N	7/85	0/50	3/50	1/40	**9/10**							
Spe	%	5.9%	0.0%	2.0%	0.0%	16.0%	**100%**						
	N	5/85	0/50	1/50	0/40	4/25	**10/10**						
Pan	%	11.8%	2.5%	5.0%	0.0%	15.0%	5.0%	**100%**					
	N	8/68	1/40	2/40	0/32	3/20	1/20	**6/6**					
Ago	%	0.0%	0.0%	0.0%	0.0%	20.0%	25.0%	56.3%	**100%**				
	N	0/68	0/40	0/40	0/32	4/20	5/20	9/16	**6/6**				
PTSD	%	3.4%	0.0%	2.1%	1.3%	1.1%	15.8%	6.6%	0.0%	**75.4%**			
	N	11/323	0/190	4/190	2/152	1/95	15/95	5/76	0/76	**129/171**			
ADHD	%	1.6%	0.0%	5.6%	0.7%	1.1%	11.1%	1.4%	0.0%	5.8%	**64.7%**		
	N	5/306	0/180	10/180	1/144	1/90	10/90	1/72	0/72	20/342	**99/153**		
Alc	%	1.4%	0.0%	1.5%	0.0%	0.0%	1.5%	3.8%	0.0%	1.2%	2.1%	**73.1%**	
	N	3/221	0/130	2/130	0/104	0/65	1/65	2/52	0/52	3/247	5/234	**57/78**	
Nic	%	2.5%	0.0%	1.4%	0.0%	2.9%	8.6%	3.6%	0.0%	3.8%	6.3%	9.9%	**100%**
	N	3/119	0/70	1/70	0/56	1/35	3/35	1/28	0/28	5/133	8/126	9/91	**21/21**
***All other diagnoses***	***%***	***3*.*7%***	***1*.*1%***	***3*.*3%***	***1*.*7%***	***4*.*2%***	***7*.*5%***	***6*.*9%***	***3*.*8%***	***2*.*9%***	***2*.*8%***	***1*.*6%***	***3*.*7%***
	***N***	***75/2040***	***13/1200***	***40/1200***	***16/960***	***25/600***	***45/600***	***33/480***	***18/480***	***66/2280***	***61/2160***	***25/1560***	***31/840***

MDE = Major depressive episode; Dys = Dysthymia; Man = Mania or hypomania; GAD: Generalised anxiety disorder; Soc = Social phobia; Spe = Specific phobia; Pan = Panic disorder; Ago = Agoraphobia; PTSD = Post-traumatic stress disorder; ADHD = Attention-deficit/hyperactivity disorder; Alc = Alcohol abuse or dependence; Nic = Nicotine dependence. % = Percentage of the number of connections relative to the number of potential connections; N = Number of connections / number of potential connections. **Bold** = Connections within diagnoses; **bold/italic** = Connections with other diagnoses.

Before zooming in on the specific associations of symptoms within and between diagnoses, we want to share a few general observations regarding the empirical network structure. First of all, symptoms of attention-deficit/hyperactivity disorder seem to form two strongly connected subclusters of attention problems (‘1’ through ‘9’, orange) and symptoms of hyperactivity/impulsivity (‘10’ through ‘18’). In addition, the affective symptoms of post-traumatic stress disorder (‘11’ through ‘17’, purple) also seem to form a subcluster, which is distinct from the other symptoms that are more directly related to the traumatic event (‘3’ through ‘10’; e.g., recollections and distressing dreams of the event). These affective symptoms showed strong connections with symptoms of a major depressive episode. Finally, symptoms of alcohol abuse (‘1’ through ‘4’, yellow) and dependence (‘5’ through ‘13’), which were two distinct diagnoses in the DSM-IV, form a single cluster.

### Associations of symptoms within diagnoses

Symptoms of generalised anxiety disorder, specific phobia, panic disorder, agoraphobia and nicotine dependence were connected to all symptoms within their diagnosis (see Table 1). The percentages of connections were lower for other diagnoses, and were lowest within attention-deficit/hyperactivity disorder (i.e., 64.7% of all potential connections) and a major depressive episode (i.e., 68.4% of all potential connections).

S2 Table provides more detailed information about the connections of each of the symptoms with all other symptoms within their diagnosis. These symptoms generally showed numerous dense connections but the number of connections differed across symptoms. For example, the (hypo)manic symptom of elevated mood (‘1’, dark grey) was connected to all nine other (hypo)manic symptoms, whereas inflated self-esteem or grandiosity (‘3’) was connected to only seven (hypo)manic symptoms. In addition, connection strengths varied considerably across symptoms, and some symptoms even showed a negative association. For example, the (hypo)manic symptom of elevated mood (‘1’) was strongly related to being more talkative (‘5’) and, to a lesser extent, psychomotor agitation (‘9’), but showed a negative association with irritable mood (‘2’). It is important to note that symptoms of disaggregated criteria also differed in their associations with other symptoms. For example, the (hypo)manic symptom of elevated mood (‘1’) was strongly related to inflated self-esteem or grandiosity (‘3’), whereas an irritable mood (‘2’) was not. Similarly, the major depressive episode symptom of insomnia (‘5’) was related to psychomotor agitation (‘7’) but not retardation (‘8’), whereas hypersomnia (‘6’) was only related to psychomotor retardation (‘8’) and not agitation (‘7’).

### Associations of symptoms between diagnoses

All diagnoses were connected to at least three other diagnoses and this number was highest for a major depressive episode and a panic disorder, which both showed connections to ten other diagnoses (see Table 1). The percentages of connections with other diagnoses were lowest for dysthymia (i.e., 1.1% of all potential connections) and alcohol abuse or dependence (i.e., 1.6% of all potential connections) and highest for specific phobia (i.e., 7.5% of all potential connections) and panic disorder (i.e., 6.9% of all potential connections).

S2 Table provides more detailed information about the connections of each of the symptoms with all other symptoms of other diagnoses. In general, overlapping symptoms were strongly connected; for example, major depressive episode and generalised anxiety disorder were mainly connected via overlapping symptoms such as sleep disturbances (‘5’ [white] and ‘8’ [light blue], respectively) and concentration problems (‘12’ and ‘5’, respectively). However, non-overlapping symptoms also showed connections; for example, we found a connection between low mood and anxiety/worry (‘1’ and ‘1’, respectively).

### Sensitivity analyses (dealing with skip-related missingness)

The AUDADIS-IV includes screening questions for all psychiatric diagnoses, except for attention-deficit/hyperactivity disorder. In our sample, these skip-related missingness ranged from 0% (*N* = 50; in participants who had a positive response to all screening questions) to 72% (*N* = 2,429; in participants who had a negative response to all screening questions). In total, the dataset contained 49% skip-related missing data.

As implied by the skip logic, the skip-related missing values on the non-screening questions were imputed with zeros, indicating absence. This imputation strategy may have artificially induced strong connections within diagnoses and weak or absent connections between diagnoses. We, therefore, wanted to repeat our analyses in the 50 participants who had full data on all symptoms (i.e., the 0.1% of our original sample who answered ‘yes’ to all screening questions). As the imputation strategy is mainly problematic for the 45 symptoms of a major depressive episode, dysthymia, mania or hypomania, and generalised anxiety disorder (see Materials and Methods section), we also wanted to repeat our analyses in the 266 participants who had full data on these symptoms (i.e., the 0.8% of the original sample who answered ‘yes’ to the screening questions of these four diagnoses). Unfortunately, both samples were not sufficiently large to estimate the network model and the high prevalence rates of multiple symptoms (e.g., rates of the screening questions were, for example, 100%) resulted in ceiling effects that severely disturbed the network estimations.

We, therefore, examined a subset of participants who had less than 20% missing data (N = 985; 2.8% of the original sample). The resulting network is presented in S1 Fig and had a largely similar segregated structure (i.e., strong connections within diagnoses and weak or absent connections between diagnoses) as our original network (Fig 1).

## Discussion

Our study was the first to apply a network analysis technique to a broad range of DSM-IV symptoms in a large community sample. The empirically based network structure corresponded with the general structure of the DSM, but individual symptoms differed substantially in their associations within the same diagnosis as well as their associations with symptoms of other diagnoses. These findings indicate that each of the psychiatric symptoms have a unique role and are, therefore, not interchangeable.

Our empirically based network structure supports the global framework of the DSM, as, in general, symptoms showed more connections to symptoms within the same diagnosis (i.e., 64.7%-100% of all potential connections) than to symptoms of other diagnoses (i.e., 1.1%-6.9% of all potential connections). Interestingly, even subtypes of, for example, attention-deficit/hyperactivity disorder were reflected in the network structure; namely, as subclusters consisting of attention problems and symptoms of hyperactivity/impulsivity. In contrast, symptoms of the DSM-IV alcohol abuse and alcohol dependence diagnoses form a single cluster, which is concordant with the recent adaptations in the DSM-5 of combining these symptoms into one diagnosis of an alcohol-use disorder. The atypical subtype within a major depressive episode was not reflected by the network structure, as, for example, hypersomnia was not only associated with an increase in weight/appetite but also with a decrease. This might be in line with a recent review of Van Loo et al. [30] who showed that studies to date provide insufficient evidence for consistent data-driven subtypes based on clusters of symptoms and advocated that research may benefit from more sophisticated statistical techniques.

Although our findings generally support the global structure of the DSM, the network also incorporates important additional information on the unique role of individual symptoms. For example, associations of symptoms within the same diagnosis differed substantially in both strength (strong, weak or absent connections) and direction (positive or negative connections). This was also true for the symptoms of disaggregated criteria. The complex constellation of associations within the same diagnosis underlines that individual symptoms are unique and not interchangeable. This is in line with previous research showing that individual symptoms have different risk factors [8,9] and consequences [11]. A strategy of summing symptoms, either for establishing broad diagnoses or for distinguishing specific subtypes within diagnoses, thus, leads to loss of information.

In addition, all diagnoses were connected via specific symptom pairs to at least three other diagnoses. This indicates that these specific connecting symptoms, and not any of the other symptoms, can directly account for the comorbidity between the two diagnoses. This is valuable information that may provide important insights into the mechanisms underlying comorbidity and that would have remained uncovered in an approach focussing on diagnoses instead of symptoms. In our network, especially overlapping symptoms were strongly related and call into question whether, for example, sleep disturbances in the context of a major depressive episode really differ from sleep disturbances in the context of a generalised anxiety disorder or post-traumatic stress disorder. In addition, non-overlapping symptoms of different diagnoses also showed strong connections; low mood (major depressive episode) was connected to, for example, worry (of generalised anxiety disorder), fear of a specific situation (specific phobia) and failure to finish things such as schoolwork and chores (attention-deficit/hyperactivity disorder). Our findings are in line with previous groundbreaking work of Cramer et al. [2], who were the first to construct the network structure of symptoms of both a major depressive episode and a generalised anxiety disorder and found connections between both overlapping and non-overlapping symptoms.

An important strength of the network analysis technique is that it can provide detailed information on the complex relations between psychiatric symptoms. As it more adequately captures the complexity of psychopathology, it may also allow us to examine the multifactorial etiology of psychopathology in all its complexity. In the last decades, etiological studies have largely been disappointing [31,32] but this may well be a consequence of the oversimplified conceptualisation of psychopathology as psychiatric diagnoses [12]. By extending our network structure of DSM-based psychiatric symptoms with, for example, genetic, pathophysiological, behavioural and psychological factors, it may be possible to determine whether specific etiological factors (e.g., cortisol) link to specific symptoms (e.g., sleep disturbances).

Previous studies on the empirical network structure of psychiatric symptoms have been highly valuable, but they have only considered symptoms of two diagnoses [2,16]. Our study was unique in including 120 psychiatric symptoms of twelve major DSM-IV diagnoses. However, information on other diagnoses, such as obsessive-compulsive disorder or schizophrenia, was not available in NESARC.

It is also important to note that symptoms were assessed with a diagnostic interview that includes screening questions for all psychiatric diagnoses, except for attention-deficit/hyperactivity disorder. As implied by the skip logic, the skip-related missing values on the non-screening questions were considered as absent symptoms. As this strategy is probably sensible for most symptoms in the network (see the extensive discussion in the Materials and Methods section), we are confident that the presented, segregated structure of the symptoms of social phobia, specific phobia, panic disorder, agoraphobia, post-traumatic stress disorder, attention-deficit/hyperactivity disorder, alcohol abuse or dependence, and nicotine dependence reflects the “true network”. However, the imputation strategy is questionable for symptoms of major depressive episode, dysthymia, mania or hypomania, and generalised anxiety disorder and may have artificially induced strong connections within the diagnoses and weak or absent connections between the diagnoses. Although we have conducted a sensitivity analysis in a subsample of participants who had less than 20% missing data and reproduced the segregated network structure, this may still have introduced bias. We, therefore, strongly encourage other researchers to apply our network techniques to diagnostic information that is not assessed by instruments that follow the skip-structure of the DSM. It would also be interesting to incorporate information on behaviours, thoughts and emotions that are outside the scope of the DSM, including factors that might have a beneficial effect on mental health.

Another important limitation of our study is that information about psychiatric symptoms was based on a cross-sectional assessment with different timeframes (i.e., in the past three years, during childhood and lifetime). Consequently, it was not possible to draw conclusions regarding the temporal relationship between symptoms and we could, therefore, only present an undirected network structure. Innovative time-series analyses based on data of multiple assessments with short time intervals could help to reveal the structure of directed symptom networks. For example, recent studies have demonstrated the causal interplay of affective symptoms over time [17,18]. Similarly, symptoms of different diagnoses have also shown to be causally related, as a recent study found that increases in negative affect resulted in increases in paranoia over the subsequent three hours [19]. Furthermore, it is interesting to note that clinicians also view psychopathology as dynamic networks of causally related symptoms [12]. These models come close to clinical reality and their therapeutic application has already shown to be successful in the treatment of depression [33].

In conclusion, current classification systems, such as the DSM or ICD, have been highly valuable in clinical practice. However, psychiatric diagnoses are, by definition, simplified models of psychopathology and may not be optimal for all research purposes. The network approach has shown to be promising, as it more adequately captures the complexity of psychopathology, but more research is needed.

## Supporting Information

S1 Table Symptoms of the twelve psychiatric diagnoses. ‘#’ refers to the number in nodes of Fig 1; ‘Criterion’ refers to the criterion in DSM-IV.
(DOCX)

S2 Table Estimated connections strengths (bs) between all individual symptoms. (XLSX)

S1 Fig Empirical network of 120 psychiatric symptoms in the subsample of participants who had less than 20% missing data (N = 985). (TIFF)
