# Supplementary material for: The Network Structure of Symptoms of the Diagnostic and Statistical Manual of Mental Disorders
Source: PLoS One. 2015 Sep 14;10(9):e0137621. doi: 10.1371/journal.pone.0137621 (PMC4569413; doi:10.1371/journal.pone.0137621)
Supplement: S1 Table — ‘#’ refers to the number in nodes of Fig 1; ‘Criterion’ refers to the criterion in DSM-IV. (DOCX) [file pone.0137621.s001.docx]

| **Major depressive episode (white)** | | | | |
| --- | --- | --- | --- | --- |
| **#** | **Criterion** | **Description** | **N** | **%** |
| *Screening question* | | Low mood (**1**) or loss of interest (**2**) | 7,400 | 21.4 |
| **1** | A1 | Low mood | 6,718 | 19.4 |
| **2** | A2 | Loss of interest | 5,349 | 15.4 |
| **3** | A3 | Decrease in weight/appetite | 2,863 | 8.3 |
| **4** | A3 | Increase in weight/appetite | 2,626 | 7.6 |
| **5** | A4 | Insomnia | 4,417 | 12.7 |
| **6** | A4 | Hypersomnia | 2,248 | 6.5 |
| **7** | A5 | Psychomotor agitation | 2,581 | 7.4 |
| **8** | A5 | Psychomotor retardation | 2,036 | 5.9 |
| **9** | A6 | Fatigue | 4,728 | 13.6 |
| **10** | A7 | Feelings of worthlessness | 2,499 | 7.2 |
| **11** | A7 | Feelings of guilt | 2,696 | 7.8 |
| **12** | A8 | Diminished ability to concentrate | 4,314 | 12.4 |
| **13** | A8 | Indecisiveness | 3,691 | 10.7 |
| **14** | A9 | Thoughts of death | 1,859 | 5.4 |
| **15** | A9 | Suicidal ideation without plan | 1,971 | 5.7 |
| **16** | A9 | Suicidal ideation with plan | 1,284 | 3.7 |
| **17** | A9 | Suicide attempt | 241 | 0.7 |
| **Dysthymia (light grey)** | | | | |
| **#** | **Criterion** | **Description** | **N** | **%** |
| *Screening question* | | Low mood for at least 2 years (**1**) | 968 | 2.8 |
| **1** | A | Low mood for at least 2 years | 968 | 2.8 |
| **2** | B1 | Decrease in appetite | 529 | 1.5 |
| **3** | B1 | Increase in appetite | 393 | 1.1 |
| **4** | B2 | Insomnia | 729 | 2.1 |
| **5** | B2 | Hypersomnia | 463 | 1.3 |
| **6** | B3 | Fatigue | 857 | 2.5 |
| **7** | B4 | Low self-esteem | 775 | 2.2 |
| **8** | B5 | Diminished ability to concentrate | 793 | 2.3 |
| **9** | B5 | Indecisiveness | 741 | 2.1 |
| **10** | B6 | Feelings of hopelessness | 757 | 2.2 |
| **Mania or hypomania (dark grey)** | | | | |
| **#** | **Criterion** | **Description** | **N** | **%** |
| *Screening question* | | Elevated mood (**1**) or irritable mood (**2**) | 3,349 | 9.7 |
| **1** | A | Elevated mood | 1,995 | 5.8 |
| **2** | A | Irritable mood | 1,945 | 5.6 |
| **3** | B1 | Inflated self-esteem or grandiosity | 428 | 1.2 |
| **4** | B2 | Decreased need for sleep | 1,127 | 3.3 |
| **5** | B3 | More talkative or pressure to keep talking | 1,738 | 5.0 |
| **6** | B4 | Flight of ideas or racing thoughts | 1,410 | 4.1 |
| **7** | B5 | Distractibility | 1,659 | 4.8 |
| **8** | B6 | Increase in goal-directed behavior | 1,486 | 4.3 |
| **9** | B6 | Psychomotor agitation | 1,529 | 4.4 |
| **10** | B7 | Increase in pleasurable activities | 1,088 | 3.1 |
| **Generalized anxiety disorder (light blue)** | | | | |
| **#** | **Criterion** | **Description** | **N** | **%** |
| *Screening question* | | Anxiety or worry for at least 6 months | 2,666 | 7.7 |
| **1** | A | Anxiety or worry for at least 6 months about 2+ things | 2,063 | 6.0 |
| **2** | B | Difficulty to control the worry | 2,128 | 6.1 |
| **3** | C1 | Restlessness | 1,850 | 5.3 |
| **4** | C2 | Easily fatigued | 1,902 | 5.5 |
| **5** | C3 | Difficulty concentrating or mind going blank | 2,085 | 6.0 |
| **6** | C4 | Irritability | 1,818 | 5.2 |
| **7** | C5 | Muscle tension | 1,633 | 4.7 |
| **8** | C6 | Sleep disturbance | 1,868 | 5.4 |
| **Social phobia (dark blue)** | | | | |
| **#** | **Criterion** | **Description** | **N** | **%** |
| *Screening question* | | Fear/avoidance of social situation (**1**) | 2,474 | 7.1 |
| **1** | A | Fear/avoidance of social situation | 2,474 | 7.1 |
| **2** | B | Social situation provokes anxiety | 1,712 | 4.9 |
| **3** | C | Fear is excessive or unreasonable | 1,610 | 4.6 |
| **4** | D | Avoidance of social situations | 1,105 | 3.2 |
| **5** | D | Social situations are endured with intense anxiety | 1,586 | 4.6 |
| **Specific phobia (pink)** | | | | |
| **#** | **Criterion** | **Description** | **N** | **%** |
| *Screening question* | | Fear of specific situation (**1**) | 12,172 | 35.1 |
| **1** | A | Fear of specific situation | 12,172 | 35.1 |
| **2** | B | Specific situation provokes anxiety | 7,094 | 20.5 |
| **3** | C | Fear is excessive or unreasonable | 5,692 | 16.4 |
| **4** | D | Avoidance of specific situation | 5,207 | 15.0 |
| **5** | D | Specific situation is endured with intense anxiety | 5,001 | 14.4 |
| **Panic disorder (light green)** | | | | |
| **#** | **Criterion** | **Description** | **N** | **%** |
| *Screening question* | | Unexpected panic attack with at least 4 symptoms (**1**) | 2,713 | 7.8 |
| **1** | A1 | Unexpected panic attack with at least 4 symptoms | 2,713 | 7.8 |
| **2** | A2-A | Worried a lot about another attack | 1,212 | 3.5 |
| **3** | A2-B | Worried about consequences | 1,058 | 3.1 |
| **4** | A2-C | Made changes in everyday life | 1,220 | 3.5 |
| **Agoraphobia (dark green)** | | | | |
| **#** | **Criterion** | **Description** | **N** | **%** |
| *Screening question* | | Fear of specific situation, as assessed for specific phobia (**1**) | 12,172 | 35.1 |
| **1** | A | Afraid of having panic attack or being embarrassed or unable to find help in case of a panic attack | 372 | 1.1 |
| **2** | B | Avoidance of situation because of fear or panic attack | 454 | 1.3 |
| **3** | B | Situation is endured with worry about a panic attack | 578 | 1.7 |
| **4** | B | Situation requires the presence of a companion | 362 | 1.0 |
| **Post-traumatic stress disorder (purple)** | | | | |
| **#** | **Criterion** | **Description** | **N** | **%** |
| *Screening question* | | Exposure to traumatic event (**1**) or intense fear, helplessness or horror (**2**) | 23,860 | 68.9 |
| **1** | A1 | Exposure to traumatic event | 15,222 | 43.9 |
| **2** | A2 | Intense fear, helplessness or horror | 21,105 | 60.9 |
| **3** | B1 | Recollections of the event | 16,939 | 48.9 |
| **4** | B2 | Distressing dreams of the event | 7,848 | 22.6 |
| **5** | B3 | Acting or feeling as if the event were recurring | 7,556 | 21.8 |
| **6** | B4 | Intense psychological distress | 9,565 | 27.6 |
| **7** | B5 | Physiological reactivity | 4,617 | 13.3 |
| **8** | C1 | Efforts to avoid thoughts, feelings or conversations | 12,119 | 35.0 |
| **9** | C2 | Efforts to avoid activities, places or people | 4,047 | 11.7 |
| **10** | C3 | Inability to recall an important aspect of the trauma | 3,549 | 10.2 |
| **11** | C4 | Diminished interest or participation in activities | 4,836 | 14.0 |
| **12** | C5 | Feeling of detachment or estrangement from others | 4,368 | 12.6 |
| **13** | C6 | Restricted range of affect | 2,893 | 8.3 |
| **14** | C7 | Sense of a foreshortened future | 1,727 | 5.0 |
| **15** | D1 | Difficulty falling or staying asleep | 6,806 | 19.6 |
| **16** | D2 | Irritability or anger | 4,043 | 11.7 |
| **17** | D3 | Difficulty concentrating | 6,318 | 18.2 |
| **18** | D4 | Hyper vigilance | 7,280 | 21.0 |
| **19** | D5 | Exaggerated startle response | 3,834 | 11.1 |
| **Attention-deficit/hyperactivity disorder (orange)** | | | | |
| **#** | **Criterion** | **Description** | **N** | **%** |
| *Screening question* | | NA | NA | NA |
| **1** | A1-A | Fails to give close attention or making careless mistakes | 3,317 | 9.6 |
| **2** | A1-B | Difficulty sustaining attention in tasks or play activities | 2,473 | 7.1 |
| **3** | A1-C | Does not seem to listen | 4,368 | 12.6 |
| **4** | A1-D | Fails to finish schoolwork, chores, etcetera | 5,655 | 16.3 |
| **5** | A1-E | Difficulty organizing tasks and activities | 2,173 | 6.3 |
| **6** | A1-F | Avoids, dislikes or is reluctant to engage in tasks | 4,715 | 13.6 |
| **7** | A1-G | Loses things necessary for tasks or activities | 2,248 | 6.5 |
| **8** | A1-H | Easily distracted | 4,007 | 11.6 |
| **9** | A1-I | Forgetful in daily activities | 2,196 | 6.3 |
| **10** | A2-A | Fidgets with hands or feet or squirms in seat | 4,553 | 13.1 |
| **11** | A2-B | Leaves seat when not supposed to | 2,448 | 7.1 |
| **12** | A2-C | Very active when not supposed to be | 5,579 | 16.1 |
| **13** | A2-D | Difficulty playing or engaging in activities quietly | 2,835 | 8.2 |
| **14** | A2-E | “On the go” or restless | 11,058 | 31.9 |
| **15** | A2-F | Talks excessively | 7,219 | 20.8 |
| **16** | A2-G | Blurts out answers | 4,329 | 12.5 |
| **17** | A2-H | Often has difficulty awaiting turn | 2,115 | 6.1 |
| **18** | A2-I | Often interrupt or intrudes on others | 3,812 | 11.0 |
| **Alcohol abuse (A) or dependence (D) (yellow)** | | | | |
| **#** | **Criterion** | **Description** | **N** | **%** |
| *Screening question* | | Alcohol use (i.e., at least 1 alcoholic drink in the past three years) | 22,879 | 66.0 |
| **1** | A1 | Failure to fulfill major role obligations (A) | 381 | 1.1 |
| **2** | A2 | Hazardous alcohol use (A) | 2,109 | 6.1 |
| **3** | A3 | Legal problems (A) | 384 | 1.1 |
| **4** | A4 | Social or interpersonal problems (A) | 802 | 2.3 |
| **5** | 1A | Tolerance: drink more (D) | 1,243 | 3.6 |
| **6** | 1B | Tolerance: less effect (D) | 2,351 | 6.8 |
| **7** | 2A | Withdrawal with at least 2 symptoms (D) | 1,560 | 4.5 |
| **8** | 2B | Drinking to avoid or relieve withdrawal symptoms (D) | 938 | 2.7 |
| **9** | 3 | Drinking more or longer than intended (D) | 4,070 | 11.7 |
| **10** | 4 | Desire or unsuccessful attempt to stop or control use (D) | 3,630 | 10.5 |
| **11** | 5 | Spent lot of time drinking or recovering of aftereffects (D) | 1,051 | 3.0 |
| **12** | 6 | Important activities are given up or reduced (D) | 323 | 0.9 |
| **13** | 7 | Drinking despite psychological or physical problems (D) | 1,175 | 3.4 |
| **Nicotine dependence (red)** | | | | |
| **#** | **Criterion** | **Description** | **N** | **%** |
| *Screening question* | | Nicotine use (100+ cigarette, 50+ cigar, 50+ pipe, 20+ snuff, or 20+ chewing tobacco in the past three years) | 8,289 | 23.9 |
| **1** | 1A | Tolerance: use more | 784 | 2.3 |
| **2** | 2A | Withdrawal with at least 2 symptoms | 1,774 | 5.1 |
| **3** | 2B | Use to avoid withdrawal symptoms | 1,452 | 4.2 |
| **4** | 3 | Use more than intended | 2,645 | 7.6 |
| **5** | 4 | Desire or unsuccessful attempt to stop or control use | 6,559 | 18.9 |
| **6** | 6 | Important activities are given up | 884 | 2.6 |
| **7** | 7 | Using despite psychological or physical problems | 5,471 | 15.8 |
