# Supplementary figures and images for: The Network Structure of Symptoms of the Diagnostic and Statistical Manual of Mental Disorders
Source: PLoS One. 2015 Sep 14;10(9):e0137621. doi: 10.1371/journal.pone.0137621 (PMC4569413; doi:10.1371/journal.pone.0137621)

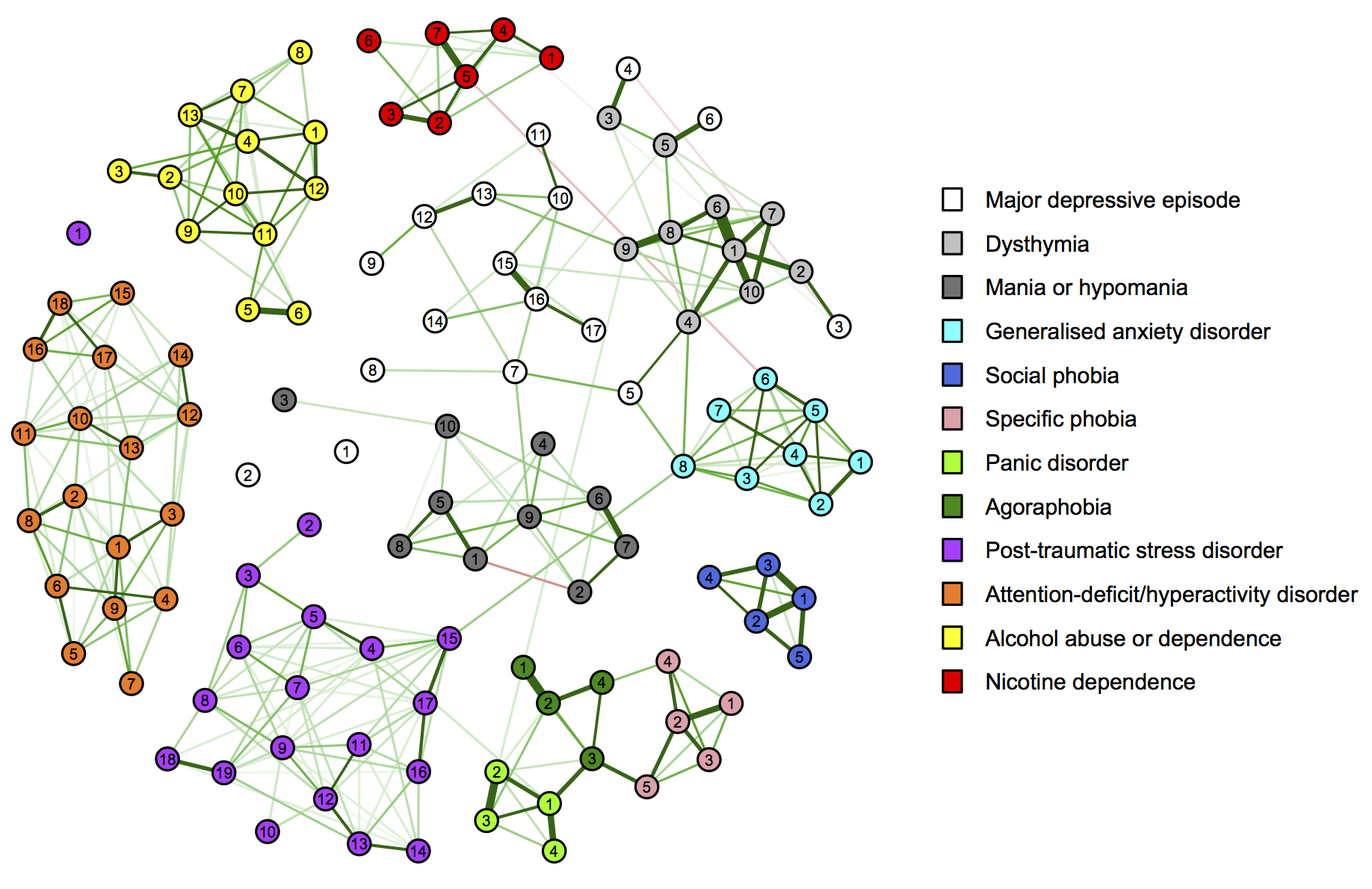

Supplement: S1 Fig — (TIFF) [file pone.0137621.s003.tiff]
